# Arctic-like canine distemper virus in partially immunized puppies

**DOI:** 10.3389/fvets.2026.1824872

**Published:** 2026-05-14

**Authors:** Zsófia Lanszki, Krisztián Bányai, Ágota Ábrahám, Brigitta Zana, Gábor Kemenesi, Kálmán Imre, Viorel Herman, János Degi

**Affiliations:** 1 National Laboratory of Virology, Szentágothai Research Centre, University of Pécs, Pécs, Hungary; 2 Faculty of Sciences, Institute of Biology, University of Pécs, Pécs, Hungary; 3 Department of Medical Biology, Medical School, University of Pécs, Pécs, Hungary; 4 Department of Pharmacology and Toxicology, University of Veterinary Medicine, Budapest, Hungary; 5 Faculty of Veterinary Medicine, University of Life Sciences “King Mihai I” from Timişoara, Timisoara, Romania

**Keywords:** Arctic-like lineage, *Morbillivirus*, *Morbillivirus canis*, Oxford Nanopore Technologies, *Paramyxoviridae*

## 1 Introduction

Canine distemper virus (CDV) is a highly contagious viral disease that poses a significant threat to domestic dogs and a wide range of wild carnivores globally (1). CDV manifests through a variety of clinical signs including respiratory, gastrointestinal and neurological symptoms (2). Despite the long-term availability of effective preventive tools, the disease remains a leading cause of mortality in canid hosts worldwide, with accumulating number of reports indicating a re-emergence and increased activity of the virus in various regions (3). The ability of the virus to cross species barriers further complicates its ecological importance and emphasizes the need for continuous surveillance in both domestic and wildlife populations (4, 5).

CDV is an enveloped, non-segmented, single-stranded, negative-sense RNA virus belonging to the genus *Morbillivirus*, family *Paramyxoviridae* (6). The viral genome encodes six structural proteins: nucleocapsid (N), phosphoprotein (P), matrix (M), fusion (F), hemagglutinin (H), and large polymerase (L) (6). Among these, the product of H gene is the main antigen mediating cell tropism and virus neutralization and in response to continuous immune pressure, H gene exhibits the highest degree of genetic variation that is utilized for lineage classification and phylogenetic studies (7). Based on H gene sequence heterogeneity, CDV is currently classified into several geographically distinct lineages, such as America-1 (which includes most traditional vaccine strains), America-2, Asia-1, Asia-2, and the Arctic-like lineage (8). The Arctic-like lineage is commonly found in wild animals and has been increasingly identified in domestic dogs in large geographic regions, demonstrating a broader global distribution than previously documented (8–12).

While cases of CDV has been documented in Romania, GenBank records indicate that no sequencing data for these cases have been published to date. In one study, seven captive raccoons (*Procyon lotor*) from the Timişoara Zoological Garden were investigated. Two animals developed clinical signs and died from infection, and viral RNA was detected in all seven blood samples by RT-qPCR (13). In another study, CDV antibodies were detected in seven out of 65 dog (*Canis lupus familiaris*), resulting in a seroprevalence of 10.8% using a sandwich enzyme-linked immunosorbent assay (14). A study investigating wild carnivores reported CDV detection in 6/34 red foxes (*Vulpes vulpes*) and 2/23 golden jackals (*Canis aureus*) between 2022 and 2023 in the Timişoara region using a rapid immunochromatographic assay (15). In parts of Romania, the vaccination coverage against CDV remains low within dog populations (14).

Disease prevention primarily relies on modified live virus vaccines, which have been available for use since the 1950s (5). These vaccines are typically derived from the America-1 genotype, featuring attenuated strains such as Onderstepoort, Lederle, or Snyder Hill (1, 2). However, vaccine breakthrough events continue to occur, often in association with incomplete immunization protocols, interference from maternally derived antibodies in young puppies, or antigenic drift that allows wild-type strains to evade vaccine-induced immunity (16–18), thereby potentially leaving animals insufficiently protected and susceptible to infection. Clinical manifestations range from mild respiratory and gastrointestinal signs to severe neurological involvement, including myoclonus, seizures, ataxia, and other central nervous system disorders, particularly in young dogs (18, 19). Clinical complications in (partially) immunized animals frequently manifest as severe nervous system involvement, including acute neurologic signs such as seizures, ataxia, proprioceptive deficits, and myoclonus (18). The identification of virulent Arctic-like strains in puppies with a history of incomplete immunization underscores the importance of maintaining rigorous vaccination schedules and performing full genome sequencing to monitor the evolution of circulating variants.

## 2 Materials and methods

### 2.1 Samples collection

Brain samples taken from carcasses of two young dogs (age range, 2.5 and 3 months) that presented with clinical signs suggesting central nervous system involvement were found to be positive for CDV. Both dogs originated from Timişoara, Romania in 2023. The samples were obtained as part of routine diagnostic submissions. The puppy with ID: RO15DJUSVT had been vaccinated with a single dose of *Biocan Puppy inj. sicc. ad us. vet*. (Bioveta, a. s., Komenského, Czech Republic) at 8 weeks of age (1 mL, subcutaneously), while the puppy with ID: RO16DJUSVT had received two vaccine doses, consisting of *Biocan Puppy inj. sicc. ad us. vet* (Bioveta, a. s., Komenského, Czech Republic) at 6 weeks of age and *Biocan DHPPi inj. sicc. ad us. vet*. (Bioveta, a. s., Komenského, Czech Republic) at 8 weeks of age (1 mL, subcutaneously).

### 2.2 Viral RNA extraction and RT-qPCR

The total RNA was extracted using the Direct-Zol RNA MiniPrep Kit (Zymo Research, USA). The samples were screened with a CDV-specific real-time RT-PCR using the Luna^®^ Universal One-Step RT-qPCR Kit (New England Biolabs, Ipswich, USA). CDV-F 5′-AGCTAGTTTCATCTTAACTATCAAATT-3′ and CDV-R 5′-TTAACTCTCCAGAAAACTCATGC-3′ primers, and CDV-Pb 5′-FAM-ACCCAAGAGCCGGATACATAGTTTCAATGC-TAMRA-3′ probe were previously published (20). The reaction setup was as follows: 55 °C for 10 min and 95 °C for 1 min, followed by 45 cycles of 95 °C for 10 s, 46 °C for 15 s 60 °C for 15 s (the fluorescence signal was detected during the annealing step). The test was run on the MyGo Mini PCR system platform (IT-IS Life Science, Ireland) and analyzed with the MyGo PCR software (v.3.5.2) (IT-IS Life Science, UK). The RT-qPCR assay was performed immediately after RNA extraction without freeze-thawing the nucleic acid, avoiding RNA degradation.

### 2.3 Nanopore sequencing and data analysis

The complete coding region of the genome was determined using an amplicon-based sequencing protocol that combines amplification of adjacent CDV genomic fragments with MinION nanopore sequencing (Oxford Nanopore Technologies, UK). The amplicon-based sequencing method for canine distemper virus, together with the primers employed, has been described previously (21), and the complete protocol is also available on the protocols.io page (https://www.protocols.io/view/universal-amplicon-based-sequencing-method-for-can-x54v9j6mpg3e/v1/metadata). In brief, cDNA was prepared from extracted viral RNA with Superscript IV (Invitrogen, USA) using random hexamers. Overlapping PCR products were amplified from the cDNA with the Q5 High-Fidelity DNA Polymerase (New England Biolabs) with multiple primer sets in parallel pools and then purified with AMPure XP beads (Beckman Coulter, USA). The primer sequences used were designed to generate overlapping amplicons of approximately 1,000 and 2,000 nt in size and no modifications were made to the original CDV sequencing protocol. The end-repair and dA tailing were performed with the NEBNext Ultra II End Repair/dA-Tailing Module (New England Biolabs). DNA barcodes from SQK-NBD114.96 (Oxford Nanopore Technologies) were ligated with Blunt/TA Ligase Master Mix (New England Biolabs, USA). After purification with Ampure XP beads (Oxford Nanopore), the NA sequencing adapters (Oxford Nanopore Technologies) were ligated with NEBNext Quick Ligation Module. The quantity of the final library was measured with Qubit dsDNA HS Assay Kit (Invitrogen, USA) on a Qubit 4 fluorometer. The final library was loaded onto a Flongle flow cell (FLO-FLG114, Oxford Nanopore Technologies) on MinION Mk1B.

The raw sequencing data were basecalled using Guppy (ONT Guppy v 6.5.7) superaccurate basecaller algorithm (dna_r10.4.1_e8.2_400bps_sup.cfg). From the fastq files, only reads with a minimum Q score of 10 were selected for the analysis which is the preset in the superaccurate model. Demultiplexing and trimming of barcodes were performed with Guppy using the guppy_barcoder enabling trimming of barcodes and setting the minimum of barcode score to 80. Read quality was checked with NanoPlot (v1.32.1) (Oxford Nanopore Technologies, UK); the overlapping primer regions were trimmed, and the reads were filtered on the expected length of amplicons with NanoFilt (v2.8.0) (Oxford Nanopore Technologies, UK). To generate a consensus sequence, the processed reads from both samples were mapped to the CDV genome (NCBI accession number: KF914669) using MiniMap2 (v2.24) (open-source software) and polished with Medaka (v2.0.1) (Oxford Nanopore Technologies, UK). The generated consensus sequences were manually checked for basecalling errors.

### 2.4 Phylogenetic and recombination analysis

In the phylogenetic analysis, 108 near-complete genome and 63 full-length H gene sequences were included. Prior to the phylogenetic reconstruction, cognate sequences were retrieved from the GenBank database and aligned with our sequences using the MAFFT alignment webserver (22). The final complete genome dataset represented all major CDV lineages, whereas the H gene alignment included all known Arctic-like sequences. The IQ-TREE webserver (23) was used to select substitution models and reconstruct maximum-likelihood phylogenetic trees, with 1,000 ultrafast bootstrap replicates. The phylogenetic analyses were conducted using the best-fitting model according to the Akaike Information Criterion (AIC); for the complete genome alignment and the Arctic-like H gene, these models were GTR+F+R3 and HKY+F+G4, respectively. Subsequently, the obtained trees were edited in the iTOL webserver (24). In addition, the obtained nucleotide sequences were feed to the NCBI BLASTn web tool to evaluate identities with cognate sequences (https://blast.ncbi.nlm.nih.gov/).

Complete genome sequences were used to identify potential recombination events in the two Romanian strains, using the available algorithms implemented in the Recombination Detection Program v.4.101 (RDP4) (25). Default settings were used for each algorithm.

## 3 Descriptive results

In 2023, brain samples were collected from carcasses of puppies, originally presented to veterinary clinic with neurologic symptoms. The 2.5 months of age puppy presented with seizures and neurological symptoms (ID: RO15DJUSVT) in July, whereas the 3 months of age puppy presented with ataxia and profound lethargy (ID: RO16DJUSVT) in September. The suspected CDV specific clinical diagnosis was confirmed by positive test result for CDV via real-time RT-PCR assay. The recommended three-dose immunization regimen where regular vaccination starts at 6 weeks of age with Biocan DP and continues at 9 and 12 weeks of age with different composition of multivalent vaccines. One animal received a single dose of vaccine (Biocan DP) and the other animal two doses of vaccine against CDV.

Both PCR-positive brain derived RNA samples were subjected to WGS using an amplicon tile sequencing protocol. This protocol was developed earlier by our research team already demonstrated its robustness and utility in epidemiologic surveillance (21, 26–28). The WGS assay relies on the enrichment of the viral genome through a multiplex PCR strategy. Specifically, it utilizes a set of overlapping primer pairs to amplify the entire genome in discrete sections, ensuring comprehensive coverage even in samples with lower viral loads. A total of 112,125 and 33,692 sequence reads were generated to assemble the two CDV genomes, achieving mean coverage depths of 11,056 × and 2,760 × for samples PX938970 and PX938971, respectively. Both assembled genomes were 15,349 nt in length, representing 97.8% of a typical CDV genome [spanning nt 199–15,547 relative to a reference genome, KX024708 (strain CDV2784/2013)]. These sequences showed high nucleotide identity with most closely related GenBank records, such as KF914669 (98.83%), KX024708 (98.79%), and PQ584613 (98.69%). Missing fragments at the 5′ and 3′ ends resulted in the loss of 91 and 37 nt in the N and L genes, respectively. Consequently, the lengths of genes and their corresponding proteins were as follows: N, 1,481 nt (incomplete N-terminus); P/C, 1,524/525 nt; 508/175 aa; M, 1,008 nt/336 aa; F, 1,989 nt/662 aa; H, 1,824 nt/608 aa; L, 6,518 nt (incomplete C-terminus).

**Figure 1:**
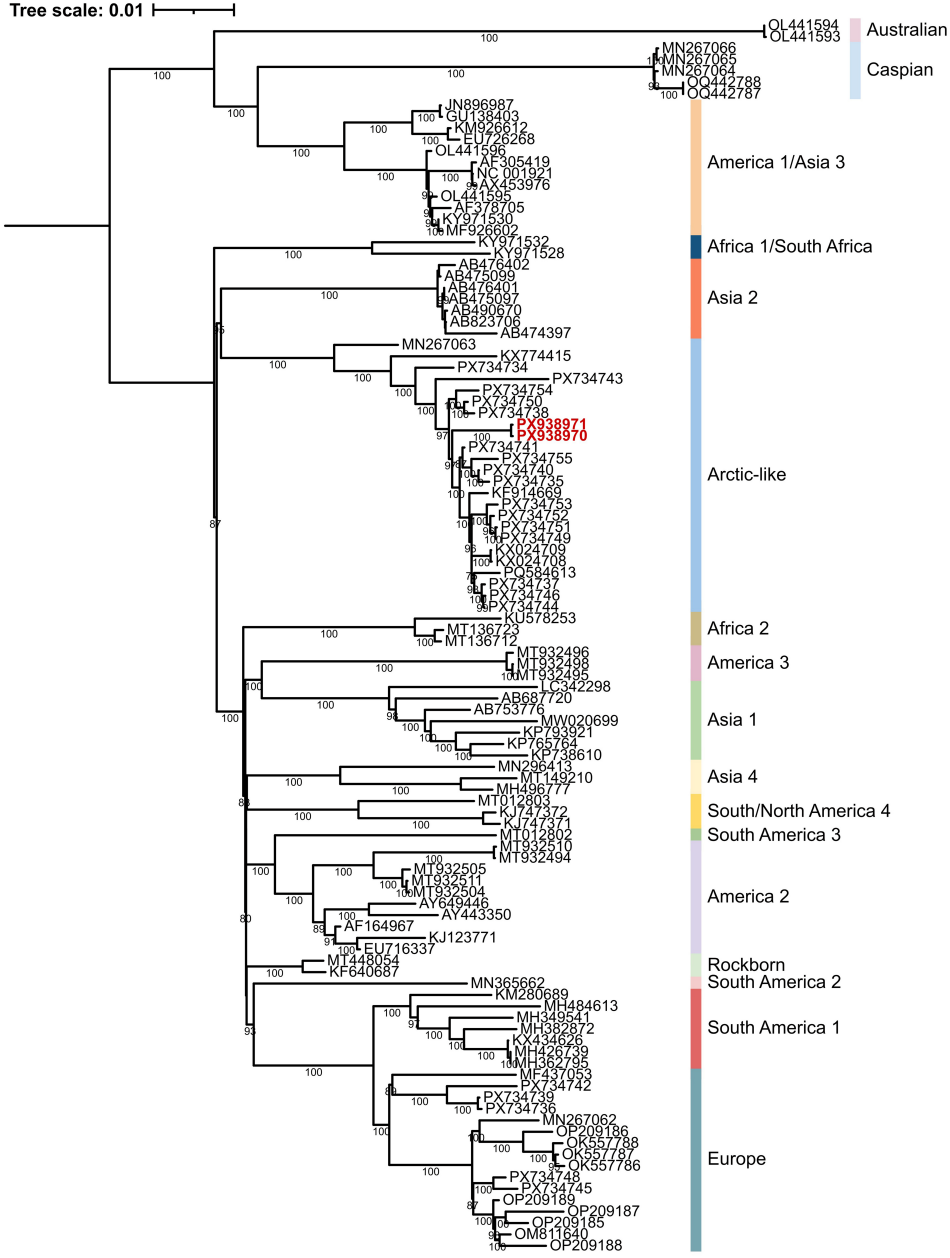
Maximum likelihood phylogenetic tree based on 108 CDV complete or near-complete genomes. The novel sequences from Romania are indicated with red color.

**Figure 2:**
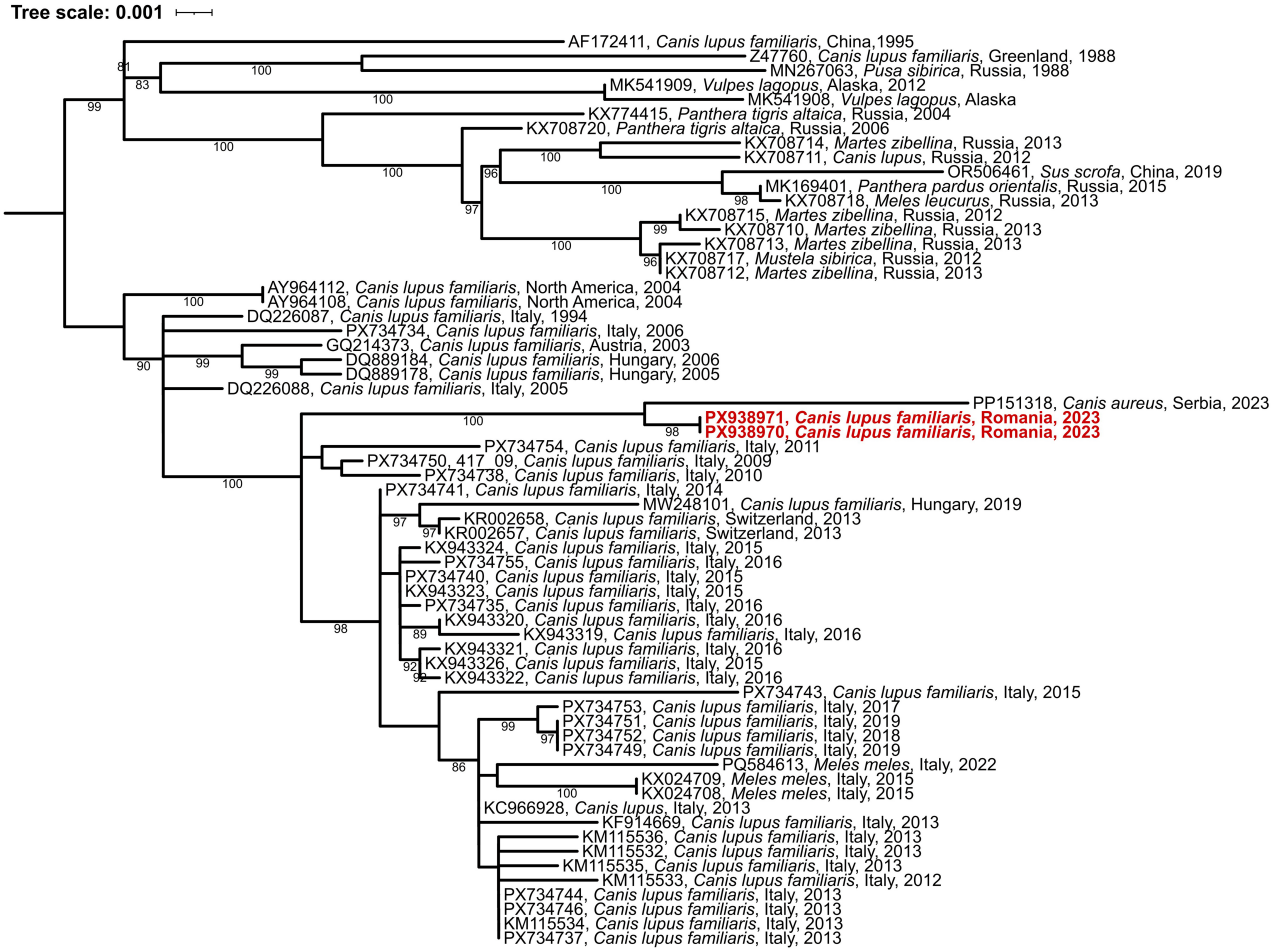
Maximum likelihood phylogenetic tree based on 63 full-length H gene sequences from Arctic-like lineage. The novel sequences from Romania are indicated with red color.

Both genome-wide and gene-specific sequence homologies exceeded 98% compared to the most closely related CDV strains, all of which were classified within the Arctic lineage. Supporting this classification, a complete genome phylogenetic analysis of representative strains from all major CDV lineages clustered the Romanian samples PX938970 and PX938970 within the Arctic-like clade (Figures 1, 2). These sequences clustered with maximum bootstrap support, indicating they are nearly identical (only 6 nt differences were detected). The phylogenetic clustering of the Romanian strains with previously reported Arctic-like CDV sequences from Europe and Asia further supports the broad geographic distribution of this lineage across multiple host species (9–12). These findings suggested the local spread of a common viral clone in the region of Timisoara. Notably, neither Romanian strains showed evidence of genomic recombination. The H gene of the study strains exhibited the highest genetic identity to a recently sequenced isolate from a Serbian golden jackal (*Canis aureus*; PP151318) (29), suggesting active viral circulation within Balkan wildlife. Furthermore, the Serbian-Romanian cluster belongs to a broader sub-clade of Italian samples from the mid-2010s that encompasses a diverse host range, including domestic dogs, wolves, and badgers (10, 30, 31). This proximity underscores the broad host range of the Arctic-like lineage and points toward a complex transmission network involving various carnivore species across Southern and Eastern Europe.

The identification of wild-type Arctic-like CDVs in partially immunized puppies highlights the significant threat posed by circulating field strains and the vulnerability of incomplete vaccination protocols. By performing WGS directly from brain tissue without viral isolation, this study provides a more accurate representation of the virus in its natural state. This approach is thought to minimize the occurrence of changes caused by adaptive evolution, such as accumulation of point mutations that frequently arise when a virus is isolated and serially passaged in cell culture before genetic characterization. Consequently, these data enable a clearer identification of key genetic markers for future genome-wide association studies on CDV neuroinvasiveness and neurovirulence.. Currently, the number of well-characterized genome sequences from brain without prior virus isolation especially from dogs is scarce, thus, our study is an important contribution toward the construction of a comprehensive reference dataset based on which genome-wide association studies can be performed. To our knowledge, these sequences represent the first two complete CDV genomes from Romania deposited in the GenBank database. High-resolution genomic insights are essential for tracking evolution of the Arctic-like lineage across Europe and for developing more effective diagnostic and preventive measures to combat severe neurological CDV infections. These findings support the importance of complete vaccination schedules in dogs and continuous molecular surveillance in both dogs and wild animals to better understand CDV epidemiology across different geographic areas.

## Data Availability

The datasets generated/analyzed for this study can be found in online repositories. The names of the repository and accession numbers can be found at: https://www.ncbi.nlm.nih.gov/genbank/, accession numbers PX938970 and PX938971.
